# Genome-Wide Association Study-Based Identification of SNPs and Haplotypes Associated With Goose Reproductive Performance and Egg Quality

**DOI:** 10.3389/fgene.2021.602583

**Published:** 2021-03-12

**Authors:** Guangliang Gao, Dengfeng Gao, Xianzhi Zhao, Songsong Xu, Keshan Zhang, Rui Wu, Chunhui Yin, Jing Li, Youhui Xie, Silu Hu, Qigui Wang

**Affiliations:** ^1^Institute of Poultry Science, Chongqing Academy of Animal Science, Chongqing, China; ^2^Institute of Animal Genetics and Breeding, College of Animal Science and Technology, Sichuan Agricultural University, Chengdu, China; ^3^Chongqing Engineering Research Center of Goose Genetic Improvement, Chongqing, China; ^4^State Key Laboratory of Agrobiotechnology, China Agricultural University, Beijing, China; ^5^Chinese Academy of Sciences, Beijing, China

**Keywords:** goose, GWAS - genome-wide association study, marker-assisted selection (MAS), egg production, egg yolk color, SNP

## Abstract

Geese are one of the most economically important waterfowl. However, the low reproductive performance and egg quality of geese hinder the development of the goose industry. The identification and application of genetic markers may improve the accuracy of beneficial trait selection. To identify the genetic markers associated with goose reproductive performance and egg quality traits, we performed a genome-wide association study (GWAS) for body weight at birth (BBW), the number of eggs at 48 weeks of age (EN48), the number of eggs at 60 weeks of age (EN60) and egg yolk color (EYC). The GWAS acquired 2.896 Tb of raw sequencing data with an average depth of 12.44× and identified 9,279,339 SNPs. The results of GWAS showed that 26 SNPs were significantly associated with BBW, EN48, EN60, and EYC. Moreover, five of these SNPs significantly associated with EN48 and EN60 were in a haplotype block on chromosome 35 from 4,512,855 to 4,541,709 bp, oriented to *TMEM161A* and another five SNPs significantly correlated to EYC were constructed in haplotype block on chromosome 5 from 21,069,009 to 21,363,580, which annotated by *TMEM161A*, *CALCR*, *TFPI2*, and *GLP1R*. Those genes were enriched in epidermal growth factor-activated receptor activity, regulation of epidermal growth factor receptor signaling pathway. The SNPs, haplotype markers, and candidate genes identified in this study can be used to improve the accuracy of marker-assisted selection for the reproductive performance and egg quality traits of geese. In addition, the candidate genes significantly associated with these traits may provide a foundation for better understanding the mechanisms underlying reproduction and egg quality in geese.

## Introduction

As one of the most economically important waterfowl, geese are widely raised for their eggs, meat, liver, feathers, and other byproducts (Boz et al., 2017). Geese are a seasonally breeding waterfowl; that is, their reproduction is regulated by natural light, and they experience a non-laying period during the year (Wang et al., 2005; Liu et al., 2020), resulting in low reproductive performance. Broodiness can further deteriorate goose reproduction, hindering the development of the goose industry (Yu J. et al., 2016).

Eggs are inexpensive source with essential vitamins and minerals (Pires et al., 2019). In an egg, the yolk is rich in various amino acids, fatty acids, vitamins, and minerals, which provide enough nutrition for the developing embryo (Zaheer, 2017; Zhu et al., 2020). The shell is also an essential component of the egg, as it provides calcium for embryonic development (Samiullah and Roberts, 2014). Additionally, the shell quality affects critical economic indexes such as embryo viability, hatchability, egg production, and the breakage rate (Yamak et al., 2016; Guoxian et al., 2017; Farghly et al., 2019). Given the importance of goose reproductive performance and egg quality, researchers focused on improving the production and quality of egg of goose (Ouyang et al., 2020; Zhao et al., 2020).

In birds, the body weight at birth, egg production and egg yolk color traits are quantitative traits, presumed to be controlled by numerous genes or loci in the genome with minor effects, and also influenced by multiple factors, including genetic factors, diet, housing, environment, management, disease, and other factors, such as environment, diet, housing practice (Dikmen et al., 2016; Mwaniki et al., 2018; Gou et al., 2020; Nemati et al., 2020; Zhao et al., 2020). Compared with other technologies, whole-genome resequencing technology is a high-throughput sequencing approach with efficient, accurate, full information characteristics (Fuentes-Pardo and Ruzzante, 2017). Moreover, whole-genome resequencing is one of the efficient and powerful strategies to screen the genes or loci in the genome widely, especially for the unknown gene and structural variation, which have been widely applied in marker-assisted selection in animals, such as the chicken (Liu et al., 2018b, 2019), duck (Rowland et al., 2018; Zhou et al., 2018), goose (Zhao et al., 2020), cattle (Freebern et al., 2020), pig (Zhang et al., 2019), and sheep (Kominakis et al., 2017).

Genome-wide association studies (GWASs) are often used to screen for genes or markers associated with specific traits (Zhang et al., 2012). To date, a number of genes or markers associated with reproductive performance, egg quality, and other economically important traits have been identified in poultry. These genes or markers could improve these economic traits through marker-assisted selection (Liu et al., 2018a; Zhou et al., 2018; Du et al., 2020). However, few genes and markers associated with the reproductive performance and egg quality of geese have been identified.

In this study, we employed a whole-genome resequencing approach to identify single nucleotide polymorphisms (SNPs) in the genome of the Sichuan goose; a GWAS analysis was then used to screen the corresponding genomic regions and candidate genes for the reproductive performance and egg quality traits. Moreover, MALDI-TOP MS method were used to verify the SNPs and haplotype blocks that are significantly associated with the traits in goose population. This work will lay a foundation for further studies on goose traits to facilitate genetic selection.

## Materials and Methods

### Ethics Statement

All experiments involving animals were performed according to the laws and regulations established by the Ministry of Agriculture of China (Beijing, China). This study was approved by the Animal Care and Welfare Committee of the Chinese Chongqing Academy of Animal Science (Chongqing, China).

### Experimental Animals and Phenotypic Traits

A total of 209 Sichuan white geese were used in this study. Each bird was reared from birth to the non-laying period (65 weeks). All individuals were housed in single cages (600 mm × 800 mm × 900 mm) at the AnFu Waterfowl Breeding Base in Chongqing City, China (105.478°N, 29.343°E). All geese were raised under natural temperatures and artificial lighting (12 h of light per day) and fed the same diet with free access to water. At week 28, blood samples were collected from the wings of all geese using vacuum tubes containing ethylenediaminetetraacetic acid. These blood samples were used for DNA extraction.

During the egg-laying period (weeks 28–64), we collected eggs four times a day and marked the eggs, which allowed us to trace the traits to the specific one in the future study. The body weight at birth (BBW) and the number of eggs at 48 weeks of age (EN48) or 60 weeks of age (EN60) were individually recorded and statistic. The three consecutive laid eggs from 209 individuals in a week during the experimental period (from 35 to 40 weeks) were used to evaluate the egg yolk color (EYC) using the Roche Yolk Color Fan (1–15: light yellow to orange) (Islam et al., 2017).

### Whole-Genome Resequencing and SNP Calling

We extracted genomic DNA from whole blood samples using a genomic DNA extraction kit (DP332; Tiangen Biotech, Beijing, China). The DNA concentration and quality were determined using a NanoVue spectrophotometer (Cytiva Life Sciences, Marlborough, MA, United States) and agarose gel electrophoresis to verify the quality requirements of the Illumina sequencing platform.

Whole-genome resequencing data were obtained using the Illumina HiSeq X Ten platform (Illumina, San Diego, CA, United States). After read filtering, the high-quality reads were mapped to the goose genome at the chromosome-level (Li et al., 2020) using BWA software (Li and Durbin, 2009). SNP calling was conducted using GATK (parameters: -R -ERC GVCF –genotyping-mode DISCOVERY –tmp-dir -I -O) software (McKenna et al., 2010).

### Quality Control and Population Structure Analysis

Quality control was conducted at both the individual and SNP levels using Plink (Purcell et al., 2007) with parameters of –geno 0.1 –mind 0.1 –maf 0.05 –hwe 0.0000001 (10^–7^). After quality control, 9,279,339 SNPs were identified of 209 individuals. Then, we performed principal component analysis (PCA) to assess goose population stratification with the application of Plink. Furtherly, the top two PCs (PC1 to PC2) were picked and plotted with R.

### GWAS and SNP-Based Haplotype Blocks

In this study, we performed GWAS assuming a random population without considering the familial relationships. The GWAS analysis between the traits (BBW, EN48, EN60, and EYC) and the SNPs was performed with genome-wide efficient mixed model association (GEMMA) software (Zhou and Stephens, 2012) using a mixed linear model as follows: *y* = *W*α + *x*β + ε, where y is the phenotypic value for all individuals, W is a covariance matrix used to control the population structure (fixed effects: PC1 and PC2), α is a vector of the corresponding coefficients including the intercept, *x* is the genotype of the SNP or haplotype marker, β is the effect size of the SNP or haplotype marker for the phenotypes, and ε is a vector of random residuals. The Wald test statistic was used to calculate the significance of the associations between the SNPs and phenotypes, while the association analysis results were corrected using Bonferroni’s correction (Threshold value was 1e^–7^).

The genotypes of the significantly associated SNPs identified by GWAS were determined by iPLEX matrix-assisted laser desorption/ionization time-of-flight mass spectrometry (MALDI-TOF MS) of the MassARRAY genotyping platform (Agena Bioscience, San Diego, CA, United States). Amplification and extension primers (Supplementary Table 1) were designed to amplify the target sequences and to hybridize and elongate the fragments at the nucleotide of interest, respectively.

For all of the traits, Manhattan plots were generated using the GAP package^1^, and quantile–quantile (QQ) plots were drawn using the qqman package^2^; both were performed in R project software. We used the GenABEL package to calculate the extent of false-positive signals in the results using the genomic inflation factor (*k*) (Karssen et al., 2016).

We assess the Linkage disequilibrium (LD) structure between significantly associate SNPs identified by MALDI-TOF MS method using Haploview software (version 4.2)^3^ (Barrett et al., 2005). Association of the SNPs and haplotypes with phenotypic data was carried out using a general linear model in JMP version 13.0 as follows: *y* = μ + *x* + ε, where *y* is the phenotypic value for each goose, μ is the population mean, *x* represents genotype, and ε is the random error. The least-squares mean of each genotype or haplotype block was then calculated, and differences between the genotypes were analyzed using the Bonferroni test.

### SNP Annotation

BEDTools software (Quinlan, 2014) was used to extract genetic information from 500-kb regions upstream and downstream of each potential SNP in the goose genome, while SNP annotation was conducted using Annovar software (Wang et al., 2010) (SnpEff, Annovar, VEP, and Oncotator). Functional annotation analysis of the candidate genes was performed using the Metascape website^4^ (Zhou et al., 2019).

## Results

### Phenotypic Description and SNP Calling

The body weight (BBW), laying performance (EN48 and EN60), and egg quality (EYC) of all 209 Sichuan white geese were recorded during the laying period (Table 1). A total of 2.896 Tb of raw whole-genome resequencing data were acquired from 209 female Sichuan white geese, with an average depth coverage of 12.44×. After filtering, 2.891 Tb of high-quality sequencing data were mapped to the goose reference genome sequence, with an average mapping rate of 98.15% (96.58–98.38%) (Supplementary Table 2). A total of 9,279,339 SNPs identified in the goose genome were used for further analysis (Supplementary Figure 1).

**TABLE 1:** The statistic of the BBW, EN48, EN60, and EYC in Sichuan White goose.

Traits	Number	Mean	Standard deviation	Minimun	Maximun	CV (%)
BBW, g	160	88.89	9.07	66	109	10.20
EN48	207	35.37	10.76	8.00	58.00	30.42
EN60	208	61.16	17.57	16.00	95.00	28.73
EYC	209	5.60	1.52	2.00	18.09	27.14

### Genome-Wide Association Study

**FIGURE 1:**
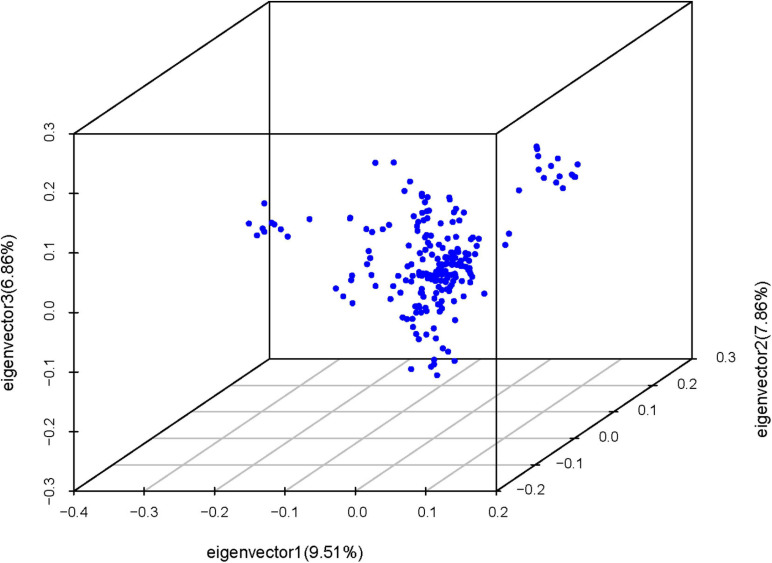
Principle component analysis for goose population using SNP markers. (The points denote individuals).

**FIGURE 2:**
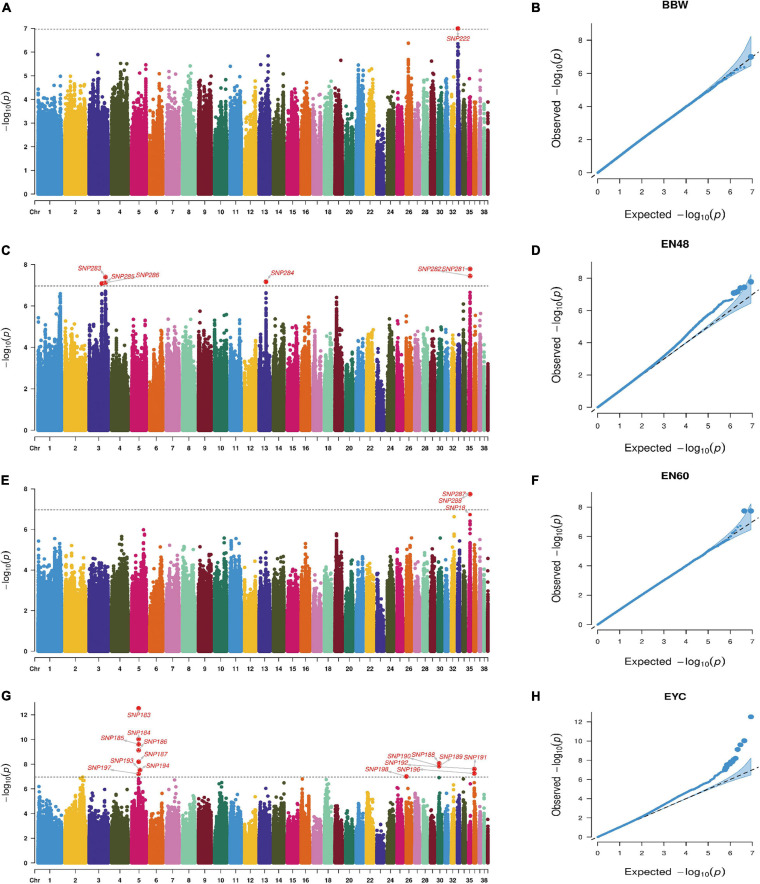
Manhattan and Q-Q plots for BBW **(A,B)**, EN48 **(C,D)**, EN60 **(E,F)**, and EYC **(G,H)** traits.

Principal component analysis revealed slight stratification within the goose population (Figure 1). Therefore, we used a mixed linear model in the GEMMA software package to adjust for variations in population structure and conducted a GWAS analysis for the selected traits. In total, 26 SNPs were found to be significantly associated with the traits (Table 2) and were located within 14 genes (Table 2). The Manhattan and QQ plots for these traits are shown in Figure 2.

**TABLE 2:** Summary of the 26 SNP markers BBW, EN48, EN60, and EYC s in goose.

SNP	Chromosome	Position (bp)	*P-*value	Traits	Location/nearest gene	Gene description
SNP16	chr35	4525296	8.29 × 10^–09^	EN64	TMEM161A	Transmembrane protein 161A
SNP17	chr35	4525312	8.29 × 10^–09^	EN64	TMEM161A	Transmembrane protein 161A
SNP222	chr33	1516391	1.01 × 10^–07^	BBW	SMAP2	Stromal membrane-associated protein 2
SNP284	chr13	20688708	6.79 × 10^–08^	EN48	PGM2L	Glucose 1,6-bisphosphate synthase
SNP283	chr3	49267770	4.03 × 10^–08^	EN48	DP13B	DCC-interacting protein 13-beta
SNP285	chr3	49260834	7.62 × 10^–08^	EN48	DP13B	DCC-interacting protein 13-beta
SNP286	chr3	38384739	8.20 × 10^–08^	EN48	RTJK	RNA-directed DNA polymerase from mobile element jockey
SNP281	chr35	4541709	1.64 × 10^–08^	EN48	TMEM161A	Transmembrane protein 161A
SNP282	chr35	4530609	3.59 × 10^–08^	EN48	TMEM161A	Transmembrane protein 161A
SNP18	chr35	4512855	1.08 × 10^–07^	EN60	S2542	Mitochondrial coenzyme A transporter SLC25A42
SNP287	chr35	4525296	8.29 × 10^–09^	EN60	TMEM161A	Transmembrane protein 161A
SNP288	chr35	4525312	8.29 × 10^–09^	EN60	TMEM161A	Transmembrane protein 161A
SNP198	chr26	443100	9.69 × 10^–08^	EYC	ZBT46	Zinc finger and BTB domain-containing protein 46
SNP188	chr30	3532845	8.33 × 10^–09^	EYC	MBB1A	Myb-binding protein 1A-like protein
SNP189	chr30	3536078	1.45 × 10^–08^	EYC	MBB1A	Myb-binding protein 1A-like protein
SNP190	chr30	3536533	1.45 × 10^–08^	EYC	MBB1A	Myb-binding protein 1A-like protein
SNP191	chr36	1514622	2.44 × 10^–08^	EYC	CB042	Uncharacterized protein C2orf42
SNP192	chr36	1514623	2.44 × 10^–08^	EYC	CB042	Uncharacterized protein C2orf42
SNP196	chr36	1510734	5.65 × 10^–08^	EYC	CB042	Uncharacterized protein C2orf42
SNP193	chr5	25366779	2.91 × 10^–08^	EYC	CACB2	Voltage-dependent L-type calcium channel subunit beta-2
SNP194	chr5	25366791	2.91 × 10^–08^	EYC	CACB2	Voltage-dependent L-type calcium channel subunit beta-2
SNP183	chr5	21235021	2.95 × 10^–13^	EYC	CALCR	Calcitonin receptor
SNP184	chr5	21235070	9.24 × 10^–11^	EYC	CALCR	Calcitonin receptor
SNP186	chr5	21249496	7.45 × 10^–10^	EYC	CALCR	Calcitonin receptor
SNP187	chr5	21363580	6.31 × 10^–09^	EYC	CALCR	Calcitonin receptor
SNP185	chr5	21232365	2.43 × 10^–10^	EYC	GLP1R	Glucagon-like peptide 1 receptor
SNP197	chr5	21069009	6.13 × 10^–08^	EYC	TFPI2	Tissue factor pathway inhibitor 2
SNP195	ctg2610	34314	3.30 × 10^–08^	EYC	CCM2	Cerebral cavernous malformations protein 2 homolog

**FIGURE 3:**
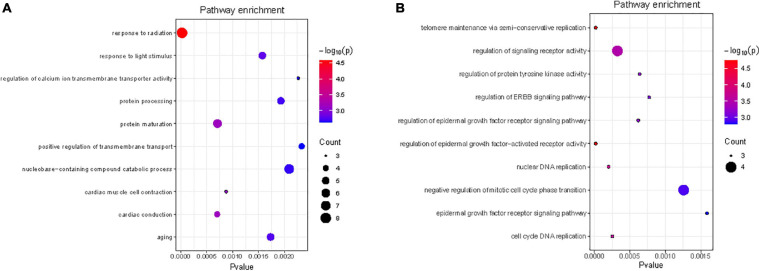
Gene ontology (GO) Biological Processes analysis for the regional candidate genes of EN48 and EN60 **(A)** and EYC **(B)** traits (top10).

We performed functional enrichment analysis for the reproductive (EN48 and EN60) and egg quality traits (EYC). The 37 Gene Ontology (GO) terms were significantly enriched (*p* < 0.05) for the EN48 and EN60 traits and included “response to radiation” (GO: 0009314, *p* = 2.05 × 10^–5^), “response to light stimulus” (GO: 0009416, *p* = 7.87 × 10^–3^), and “cellular response to hormone stimulus” (GO: 0032870, *p* = 2.16 × 10^–2^) (Supplementary Table 3 and Figure 3). The 21 GO terms were significantly enriched (*p* < 0.05) for the EYC trait, including “regulation of epidermal growth factor-activated receptor activity” (GO: 0007176, *p* = 2.00 × 10^–5^), “regulation of signaling receptor activity” (GO: 0010469, *p* = 3.20 × 10^–4^) and “regulation of epidermal growth factor receptor signaling pathway” (GO: 0042058, *p* = 6.20 × 10^–4^) (Supplementary Table 4 and Figure 3).

### Association Analysis of Haplotypes With EN48, EN60, and EYC

**FIGURE 4:**
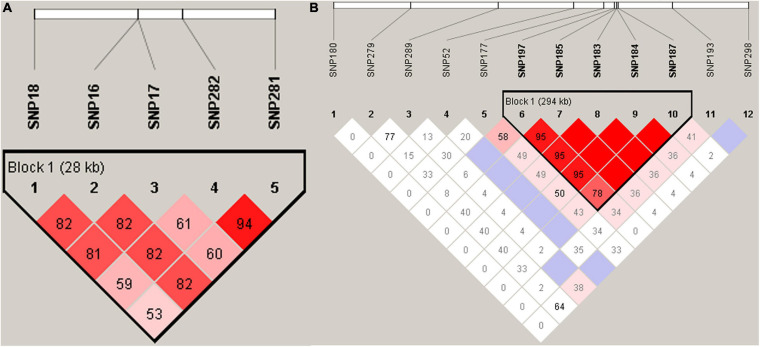
Linkage Disequilibrium (LD) analyses of SNPs in the significant region for EN48, EN60 **(A)** and EYC **(B)** traits.

Haplotypes were constructed for the regions containing the significantly associated SNPs. We found that 5 SNPs in transmembrane protein 161A (*TMEM161A*), located within a 28.85 kb genomic region (45,128,55 bp ∼ 45,417,09 bp) of chromosome 35, were significantly associated with EN48 and EN60 (Figure 4A). The 5 SNPs and genotypic distributions were statistically analyzed, and associations between genotypes and the EN48 and EN60 traits were determined (Table 3 and Figure 4A). Haplotype association analysis confirmed that this region’s corresponding haplotype was significantly associated with both the EN48 and EN60 traits. Individuals with the genotype CCAAAGAA produced fewer eggs at 48 and 60 weeks than individuals with other genotypes (Supplementary Table 5).

**TABLE 3:** Effects of the SNPs and genotypes on EYC, EN48, and EN60 traits in goose.

Traits	Genotypes (least-squares means)			*P*-value
**EYC**				
SNP183	6.86 ± 0.31^a^ (AG)	5.45 ± 0.11^a^ (AG)	/	<0.0001**
SNP184	6.86 ± 0.31^a^ (CT)	5.43 ± 0.11^b^ (TT)	/	0.0001**
SNP185	6.86 ± 0.31^a^ (GA)	5.45 ± 0.11^b^ (GG)	/	<0.0001**
SNP187	5.44 ± 0.11^b^ (GG)	6.68 ± 0.29^a^ (AG)	/	<0.0001**
SNP197	5.42 ± 0.11^b^ (GT)	6.66 ± 0.27^a^ (GG)	/	<0.0001**
**EN48**				
SNP17	36.86 ± 0.23^a^ (CC)	30.87 ± 1.67^b^ (TC)	26.71 ± 3.95^c^ (TT)	0.0019**
SNP18	36.56 ± 0.85^a^ (AA)	33.85 ± 1.81^b^ (AG)	28.42 ± 2.42^c^ (GG)	0.0049**
SNP281	37.57 ± 0.78^a^ (AA)	36.00 ± 0.99^ab^ (AG)	27.00 ± 1.51^b^ (GG)	<0.0001**
SNP282	28.25 ± 0.59^b^ (AA)	27.85 ± 1.59^b^ (AT)	37.45 ± 0.79^a^ (TT)	<0.0001**
**EN60**				
SNP17	64.55 ± 1.30^a^ (CC)	50.78 ± 2.61^b^ (TC)	43.57 ± 6.24^c^ (TT)	<0.0001**
SNP18	64.49 ± 1.34^a^ (AA)	54.26 ± 2.85^b^ (AG)	47.25 ± 3.72^c^ (GG)	<0.0001**
SNP281	66.00 ± 1.67^a^ (AA)	49.67 ± 2.54^b^ (AG)	64.14 ± 1.30^a^ (GG)	<0.0001**
SNP282	53.75 ± 0.84^b^ (AA)	50.15 ± 2.65^b^ (AT)	64.06 ± 1.31^a^ (TT)	<0.0001**

** extremely significant association P < 0.01; Different letters in the same row indicate a significant difference between the genotypes (P < 0.05); the same letter indicates no significant difference between genotypes (P > 0.05).

On chromosome 5, a haplotype within a 294.57 kb genomic region (21,069,009 ∼ 21,363,580 bp) was constructed based on 5 SNPs (Figure 4B and Table 3) in the glucagon-like peptide 1 receptor (*GLP1R*), calcitonin receptor (*CALCR*), and tissue factor pathway inhibitor 2 (*TFPI2*) genes that were significantly associated with EYC. The 5 SNPs and genotypic distributions were statistically analyzed, and associations between the genotypes and EYC traits were determined (Table 3). Haplotype association analysis showed that the haplotype was also significantly associated with EYC and that there were significant differences among the genotypes. For example, individuals with the genotypes AGCTGAGAGT and GGTTGGAAGG showed significantly higher and lower EYC values, respectively, than individuals with other genotypes (Supplementary Table 5).

## Discussion

Sichuan white goose is a popular Chinese goose breed for meat and egg purposes. The number of eggs at 60 weeks for the experimental geese were about 60 per individual, which was consistent with that described previously and laid the foundation for the further study (Xiao et al., 2010). While egg laying and egg quality traits have been studied intensively in avians, including chickens (Liu et al., 2018b, 2019), ducks (Rowland et al., 2018; Zhou et al., 2018), and quail (Wu et al., 2018), few studies have determined the associations between genetic markers and these traits (Zhao et al., 2020). In this study, we identified many SNPs and haplotypes that were associated with these traits.

There were two advantages than most of the previous studies. (1) The goose is an economically important waterfowl with distinctive characters. However, the habitat preference for water, large body size and the seasonal reproduction restricts most of the goose rearing in free-range or large groups. Compared with most of the previous studies, one of advantages in this study is that the individuals were reared in single cages (600 mm × 800 mm × 900 mm), which allowed the traits to be accurately recorded and statistically analyzed for each goose. (2) Our lab has generated a chromosome-level *de novo* assembly of the goose genome (Li et al., 2020). Our assembly has more continuity, completeness, and accuracy; the annotation of core eukaryotic genes and universal single-copy orthologs has also been improved, which significant improvement compared to previous assemblies. For example, our contig N50 value is more than fifty-fold that of prior studies. In this study, we mapped high-quality whole-genome resequencing data to the goose genome sequence and performed the GWAS analysis, which led to the results from the GWAS being more accurate than those of previous similar studies.

There was a population stratification phenomenon in the experimental goose population in this study maybe due to the limitation of the population size (209). Considering the number maybe the limitation for this study, to identify the SNPs associated with the EN48, EN60, and EYC traits, we employed the MALDI-TOF MS method to detect some selected SNP sites, and analyzed the frequency of candidate SNPs, which was consistent with the GWAS analysis results. In addition, the haplotype blocks constructed by some SNPs from GWAS analysis were also significantly associated with the traits. For instance, SNP16, SNP17, SNP18, SNP281, and SNP282 were significantly associated with the EN48 or EN60 (Supplementary Table 5), and the haplotype block (Figure 4A) constructed by the five SNPs were also significantly with the EN48 and EN60 traits (Supplementary Table 5), suggesting that the results were reliable and robust and avoided false positives.

In this study, A total of 2.896 Tb of raw whole-genome resequencing data were generated from the 209 individuals, and the average GC content was 42.85%, consistent with that previously reported for the goose genome (Lu et al., 2015; Gao et al., 2016; Li et al., 2020), which supply the robust sequencing data for the further study. A total of 26 SNPs in 209 geese were identified to be significantly associated with the BBW, EN48, EN60, and EYC traits by GWAS. As one of the most economically important traits, BBW is a crucial index of goose breeding and has been correlated with a number of other traits in birds (Sarangi et al., 2016). A SNP located within stromal membrane-associated protein 2 (*SMAP2*) was significantly associated with BBW. *SMAP2* is a member of the ArfGAP subfamily. The protein encoded by this gene exerts a vital function in vesicle trafficking and plays a crucial role in sperm formation (Natsume et al., 2006; Funaki et al., 2013). In mammals, *SMAP2* is necessary for spermiogenesis (Han et al., 2020), where *SMAP2*-deficient mice have been shown display male infertility (Sumiyoshi et al., 2015), globozoospermia (Funaki et al., 2013), asthenozoospermia (Funaki et al., 2013), and abnormal acrosome formation (Funaki et al., 2013). Therefore, we speculated that *SMAP2* might also play a key role in reproduction.

Notably, four SNPs located within *TMEM161A* were grouped into a haplotype block, and association analysis confirmed that both the SNPs and the haplotype were significantly associated with EN48 and EN60. *TMEM161A*, also known as adaptive response to oxidative stress protein 29 (*AROS-29*), is involved in several cellular processes, including the adaptive response to oxidative stress (Montesano Gesualdi et al., 2006), the response to ultraviolet radiation, and the response to retinoic acid; in addition, *TMEM161A* is a negative regulator of the intrinsic apoptotic signaling pathway in response to DNA damage and is a positive regulator of the DNA repair response. In birds, retinoic acid promotes embryonic development and egg production and can regulate the number of eggs (Fu et al., 2000; Scheider et al., 2018). Sichuan white geese display seasonal reproduction as an adaptation to the migration habits of domesticated wild geese and are therefore sensitive to UV radiation. Because *TMEM161A* plays a vital role in the response to UV light (Montesano Gesualdi et al., 2006), it is possible that the artificial selection pressures resulting from the intensification of animal reproduction systems contributed to the appearance of the four SNPs in *TMEM161A*, satisfying the demand for increased egg production and aiding in reproductive environmental adaptation. Moreover, we identified five other candidate genes for the EN48 or EN60 traits, including glucose 1,6-bisphosphate synthase (*PGM2L*), DCC-interacting protein 13-beta (*DP13B*), RNA-directed DNA polymerase from mobile element jockey (*RTJK*), and *S2542* (the mitochondrial coenzyme A transporter SLC25A42). Previous studies have identified many candidate genes corresponding to goose reproductive traits (Shigang et al., 2015; Yu S. et al., 2016; Ouyang et al., 2020; Zhao et al., 2020). However, the candidate genes for the number of eggs identified in the current study did not overlap with previous genes. The number of eggs in poultry is a quantitative trait regulated by numerous genes and environmental factors (Goto and Tsudzuki, 2016). The candidate genes and SNPs identified here were all associated with egg production despite their minor effects on the examined traits. Second, the breeds used in previous studies were raised under diverse conditions with artificial selection pressures, which would influence the different variants associated with the number of eggs trait in Sichuan white geese. Third, while the population sizes used in those studies were sufficient to identify characteristics specific to the examined breeds, they were not large enough to form a general conclusion that can be applied to all goose breeds. Finally, the goose genome sequence previous studies. For example, the value of contig N50 were improved fifty-fold than that of prior studies (Lu et al., 2015; Gao et al., 2016; Li et al., 2020). In this study, we mapped high-quality whole-genome resequencing data to the goose genome sequence and performed the GWAS analysis, which may be another reason why the GWAS results of this study were different from those of previous studies.

The pathway analysis of the candidate genes for EN48 and EN60 showed that the genes were highly enriched in response to radiation (GO: 0009314), response to a light stimulus (GO: 0009416), cellular response to a hormone stimulus (GO: 0032870), and response to a peptide hormone stimulus (GO: 0043434) (Supplementary Table 3). Geese are a seasonal breeding avian species regulated by light, and reproduction-related hormones control their reproductive activities. We speculated that the candidate genes for EN48 or EN60 were involved in the response to light or reproductive hormones, thus participating in the goose reproductive activities. However, the mechanism needs to be further studied.

Egg yolk color is a crucial economic egg quality index because consumers link yolk color to the nutrition contained in an egg (Galobart et al., 2004). Studies have shown that EYC is affected by genetic (Goto and Tsudzuki, 2016), environmental (Spada et al., 2016), housing (Sokołowicz et al., 2018), and dietary (Moreno et al., 2020) factors. In the current study, twelve SNPs were significantly associated with the EYC of Sichuan white goose eggs. Notably, five of the SNPs constituted a haplotype, leading to the identification of the candidate genes *CALCR*, *TFPI2*, and *GLP1R*. *CALCR* is a receptor that binds to the peptide hormone calcitonin and is involved in calcium homeostasis, bone formation and metabolism, and lipid metabolism (Davis et al., 2019). *GLP1R*, a member of the glucagon receptor family of G protein-coupled receptors, is involved in controlling the blood sugar level by enhancing insulin secretion (Cardoso et al., 2020). Interestingly, *CALCR* and *GLP1R* can regulate peptides with anorexic effects, thus suppressing long-term food intake and promoting significant weight loss (Yamaguchi et al., 2015; Adams et al., 2018). *TFPI-2* belongs to the Kunitz-type family of protease inhibitors and regulates matrix metalloproteinase activation and extracellular matrix degradation. Deficiency of this gene in mice accelerates atherosclerotic lesions (Hong et al., 2016). In addition, *TFPI-2* overexpression strongly inhibits vascular smooth muscle cell proliferation and migration and affects smooth muscle cell proliferation and migration (Zhao et al., 2011; Hong et al., 2016). Therefore, these positional candidate genes may control the EYC trait by affecting food intake or lipid metabolism of geese. EYC is a quantitative trait regulated by many genes in poultry (Zhang et al., 2021). In agreement with previous studies, we also identified multiple other positional candidate genes that cover the twelve SNPs, including Myb-binding protein 1A-like protein (*MBB1A*), *CB042* (known as uncharacterized protein C2orf42), voltage-dependent L-type calcium channel subunit beta-2 (*CACB2*), cerebral cavernous malformations protein 2 homolog (*CCM2*), and zinc finger and BTB domain-containing protein 46 (*ZBT46*). The effects of these genes on EYC require further study.

The GO pathway analysis revealed that the “regulation of epidermal growth factor-activated receptor activity” (GO: 0007176) and “regulation of epidermal growth factor receptor signaling pathway” (GO: 0042058) (Supplementary Table 4) were remarkably enriched for EYC traits in goose. The two GO terms are involved in modulating epidermal growth factor (EGF)-activated receptor activity. In mammals or birds, EGF-like growth factors play an essential role in the ovulatory follicle (Park et al., 2004), maturation of the cumulus-oocyte complex (Su et al., 2010), and oocyte maturation and development (Yang and Jia, 2019). Genetics is one of the critical factors affecting EYC (Mori et al., 2020). We propose that the candidate genes for the EYC trait and the EGF-activated receptor might regulate the processes of oocyte maturation and development and determine the EYC trait in geese.

## Conclusion

In the current study, a total of 26 SNPs significantly associated with egg production and quality trait were identified by GWAS. Additionally, these SNPs with significant effects were further verified using the MALDI-TOP MS method in the same population. Furthermore, 14 annotated genes were detected as candidate genes for the four traits based on their basic function. These results supplied the new candidate genetic markers and genes for the marker-assisted selection of geese, and laid the foundation for the genetic basis of the reproduction performance and egg quality traits.

## Data Availability Statement

The raw whole-genome resequencing data have been deposited in the NCBI Short Read Archive (SRA) under accession numbers: SRR10687319–SRR10687346, SRR10687348–SRR1068 7383, SRR10687385–SRR10687397, SRR10687399–SRR106874 03, SRR10687405–SRR10687410, SRR10687412–SRR10687417, SRR10687419, SRR10687421–SRR10687423, SRR10687425, SRR 10687427–SRR10687433, SRR10687436–SRR10687445, SRR106 87447–SRR10687464, SRR10687466–SRR10687473, SRR106874 75, SRR10687478, SRR10687483–SRR10687486, SRR10687488–SRR10687510, SRR10687513–SRR10687517, SRR10687519–SRR 10687528, SRR10687530, SRR10687532–SRR10687535, and SRR 10687537–SRR10687554.

## Ethics Statement

The animal study was reviewed and approved by all experiments involving animals were performed according to the laws and regulations established by the Ministry of Agriculture of China (Beijing, China). The study was approved by the Animal Care and Welfare Committee of the Chinese Chongqing Academy of Animal Science (Chongqing, China).

## Author Contributions

GG, QW, and XZ provided funds, and designed and supervised the projects. GG, XZ, JL, and YX collected the samples and the phenotypic data. GG, DG, SH, SX, and RW performed the bioinformatics analysis, data uploaded, and drawn the figures. GG, KZ, RW, and CY wrote the manuscripts. All authors contributed to the article and approved the submitted version.

## Conflict of Interest

The authors declare that the research was conducted in the absence of any commercial or financial relationships that could be construed as a potential conflict of interest. The reviewer YW declared a shared affiliation, with no collaboration, with one of the authors DG to the handling editor at the time of review.

## Footnotes

1 https://cran.r-project.org/web/packages/gap/

2 https://cran.r-project.org/web/packages/qqman/

3 http://www.broadinstitute.org/haploview

4 https://metascape.org/

## Abbreviations

AROS-29: oxidative stress protein 29
BBW: birth body weight
CACB2: voltage-dependent L-type calcium channel subunit beta-2
CALCR: calcitonin receptor
CB042: uncharacterized protein C2orf42
CCM2: cerebral cavernous malformations protein 2 homologs
EN48: the number of eggs at 48 weeks
EN60: the number of eggs at 60 weeks
GWAS: genome-wide association study
GEMMA: Genome-wide Efficient Mixed Model Association
GLP1R: glucagon-like peptide 1 receptor
LD: Linkage disequilibrium
MALDI-TOP MS: matrix-assisted laser desorption-ionization time-of-flight mass spectrometry
MBB1A: Myb-binding protein 1A-like protein
QQ plot: quantile–quantile plots
SNPs: single nucleotide polymorphisms (SNPs)
SMAP2: stromal membrane-associated protein 2
SRA: Short Read Archive
TMEM161A: transmembrane protein 161A
TFPI2: tissue factor pathway inhibitor 2
ZBT46: zinc finger and BTB domain-containing protein 46.
