# Supplementary figures and images for: Genome-Wide Association Study-Based Identification of SNPs and Haplotypes Associated With Goose Reproductive Performance and Egg Quality
Source: Front Genet. 2021 Mar 12;12:602583. doi: 10.3389/fgene.2021.602583 (PMC7994508; doi:10.3389/fgene.2021.602583)

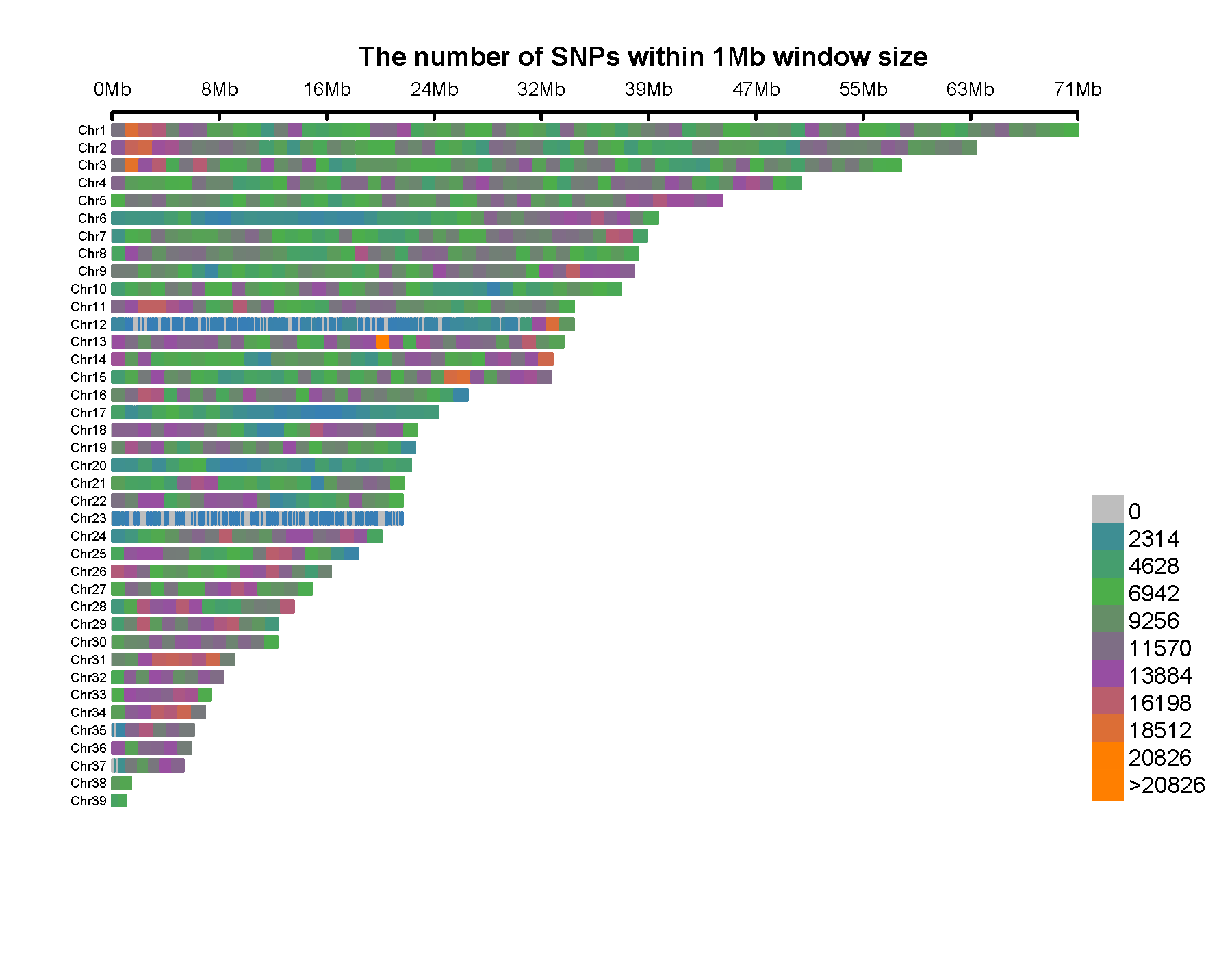

Supplement: Supplementary file 6 [file Image_1.TIFF]
